# A study of the long term changes in the electromagnetic environment using data from continuous monitoring sensors in Greece

**DOI:** 10.1038/s41598-023-41034-3

**Published:** 2023-08-23

**Authors:** Athanasios Manassas, Christos Apostolidis, Serafeim Iakovidis, Dimitrios Babas, Theodoros Samaras

**Affiliations:** 1 CIRI - Center for Interdisciplinary Research and Innovation, Aristotle University of Thessaloniki, 57001 Thermi, Greece; 2 Aristotle University of Thessaloniki, 54124 Thessaloniki, Greece

**Keywords:** Environmental sciences, Physics

## Abstract

Owing to the advancement of wireless technologies, there is a strong public perception of increasing exposure to Radiofrequency (RF) electromagnetic fields (EMF). The aim of this study is to determine the evolution of EMF in the environment, and consequently, human exposure to them, over a period of about two decades, spanning from the end of 2003 until February 2022. The study is based on data collected by two non-ionizing radiation monitoring networks in Greece. The networks consist of fixed EMF sensors that register the RMS electric field value every 6 min, on a 24 h basis. We used the Seasonal–Trend decomposition method using (LOESS), known as the STL method to decompose the time series into trend, seasonal, and noise components. Additionally, since the sensors include frequency filters for separating the cellular frequencies, the recorded data were used to identify the exposure contribution by cellular networks in comparison to other EMF sources. The study indicates that RF-EMF do not explicitly decrease or increase but rather fluctuate over time. Similarly, the contribution of mobile cellular networks to the total field change over time.

## Introduction

The advent of fifth-generation (5G) wireless technology leads anew to the deployment of network infrastructure that will serve a multitude of innovative applications (e.g., internet of things and self-driving vehicles). It is interesting how current wireless technologies affect human exposure to emitted electromagnetic fields (EMF). We are already experiencing the transition from the 4G to the 5G era. The term “transition” does not mean that innovative technologies instantaneously replace previous ones, since they exist simultaneously, in some cases for longer periods.

Mobile cellular networks have undergone significant technological advancements over the past few decades, with each generation introducing new features and capabilities. The first-generation (1G) cellular networks were developed in the 1980s and relied on analogue technology to provide voice communication. Second-generation (2G) networks introduced digital communication, which enabled data transmission in addition to voice. Third-generation (3G) networks offered higher data rates, enabling multimedia applications, and improved network capacity. Fourth-generation (4G) networks provided even faster data rates, better spectral efficiency, and advanced support for multimedia applications.

The fifth-generation (5G) cellular networks, which have recently been deployed globally, represent the latest evolution of mobile cellular networks. 5G networks are designed to offer much faster data rates, lower latency, and improved network capacity compared to previous generations. One of the key features of 5G networks is the use of millimeter-wave (mmWave) frequencies, which provide much faster data rates but have shorter ranges and can be blocked by obstacles such as buildings and trees. However, the higher frequency bands used in 5G networks also raise concerns about human exposure to electromagnetic radiation.

One of the most significant technological advancements in 5G networks is the use of beamforming and beam steering. Beamforming refers to the ability to direct a wireless signal towards a specific user or device, while beam steering allows for the dynamic adjustment of the direction of the beam to maintain a stable connection with the user or device. Beamforming and beam steering use phased array antennas, which are arrays of antenna elements that can be controlled electronically to direct the signal towards the user or device. The adoption of this technology in 5G networks is expected to improve network capacity and coverage, especially in densely populated urban areas, and offers the advantage of decreasing network interference and electromagnetic radiation emissions in unintended areas. However, it is of great interest to ascertain how the new technology will affect human exposure. This study can be used as a reference point for comparing the exposure between the pre- (2G–4G) and the post-5G era.

In Greece, the commercial application of mobile telephony started in mid-1993 with the deployment of second-generation (2G) mobile communications networks. Third-generation (3G) networks were launched in 2001. The commercial use of 4G networks began at the end of 2012 and of 5G in mid-2021, but only in a limited number of cities. It is worth mentioning that the use of 3G will be abolished by all mobile providers before the end of the first half of 2023, although 2G networks will continue to exist, mainly for long-distance wireless communications.

The transition from one generation to the next causes concerns about potential side effects on human health due to exposure to electromagnetic fields. The temporal variation of exposure to electromagnetic fields is a complex combination of many factors which are not easy to assess. Although there are several monitoring networks currently operating in Europe^1,2^, their operating period is not long enough to permit the deduction of conclusions on the impact of important technological transitions in wireless communications on the electromagnetic environment. On the other hand, the work of Rowley & Joyner^3^, based on frequency-selective and broadband measurement campaigns, was the first one to indicate that average exposure remained largely unchanged by the transition from 2 to 3G cellular networks.

The aim of this work was to evaluate the temporal evolution of environmental EMF using the recorded electric field values from the Hermes^4^ and Pedion24^5^ monitoring networks. The extensive operating period of these two networks (Hermes: December 2003 until today, Pedion24: February 2007 until today) allows us to assess the changes in exposure due to the use of 3G and 4G technologies (they operate simultaneously with 2G), as well as to identify differences during the transition from 3 to 4G.

## Materials and methods

### Sensors specifications

**Figure 1:**
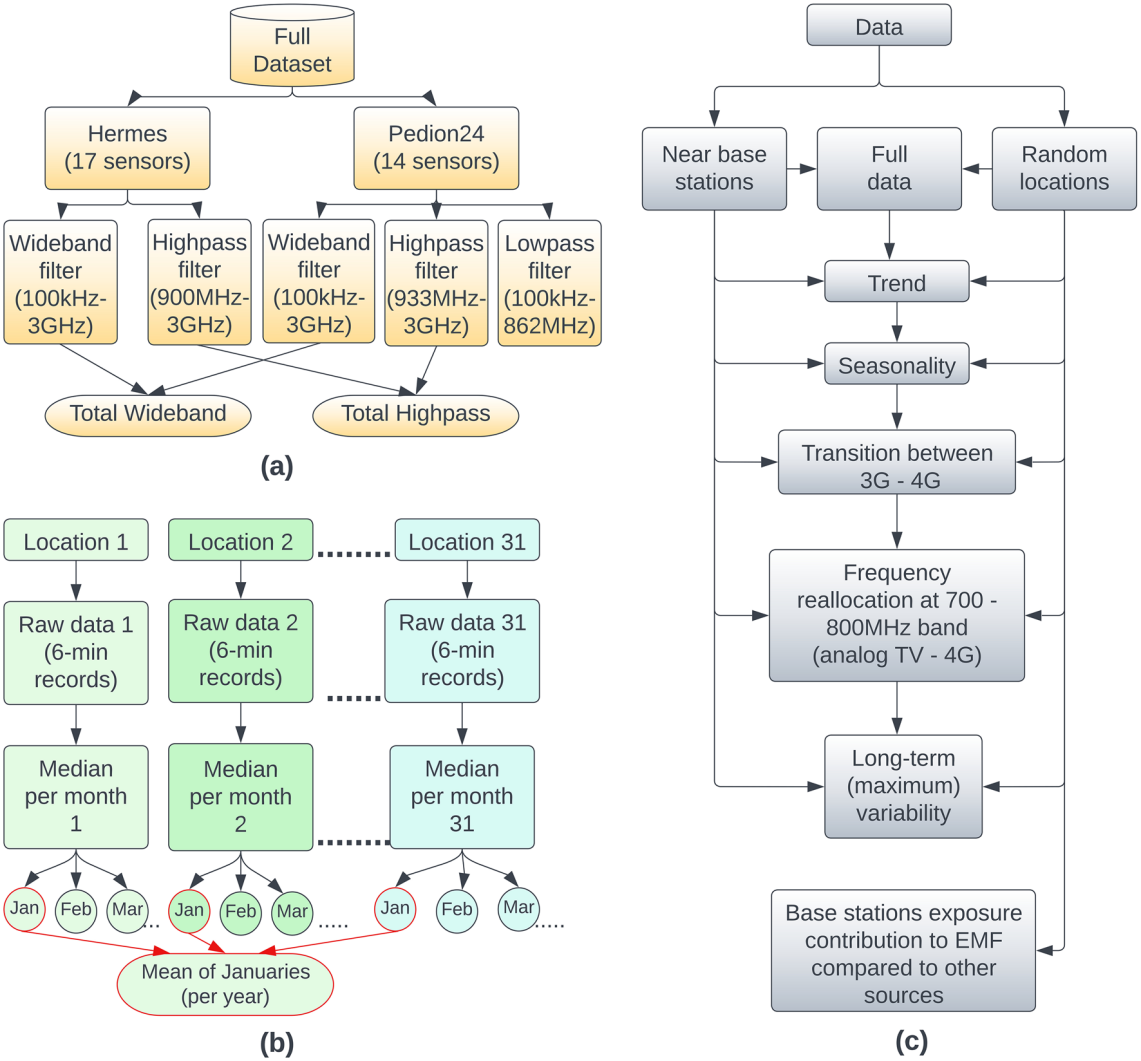
(**a**) The dataset(s) used in our analysis, (**b**) the workflow for estimating the mean value per month. The process repeated every year (2004–2022), (**c**) summary of parameters—quantities examined in this work.

The fixed monitoring sensors are equipped to continuously record the electric field 24 h a day, using tri-axial electric field probes that operate within specific frequency ranges. The monitoring sensors of the Hermes network comprise a wideband (100 kHz–3 GHz) and a high-pass (900 MHz–3 GHz) broadband filter. The sensors of Pedion24 include the same filters as above (i.e., wideband: 100 kHz–3 GHz; high-pass: 933 MHz–3 GHz) plus a low-pass filter in the frequency range of 100 kHz–862 MHz (Fig. 1a). These frequency bands cover a wide range of wireless applications, including cellular networks, FM and TV broadcasting, and RF equipment used by first responders. Additionally, the sensors are equipped with a solar panel for power supply and a GSM modem for transmitting recorded data to a server. It is important to note that the modem only operates once a day for a brief period to send the data. Any data recorded during this transmission time are disregarded because the sensors also record the field of their own modem. This duration typically lasts for only 6 min, resulting in just a single record being rejected per day. The sensors used for the Hermes network deployment and utilized in this work include the MCE-410 model (E.I.T. s.r.l, Roma, Italy). For the Pedion24 network, the sensors employed are the PMM8057 with the EP-3B-01 probe (Narda STS, Pfullingen, Germany). Table 1 includes the main technical specifications and measurement uncertainties of the monitoring sensors.

**Table 1:** Specifications of fixed monitoring sensors^6^.

Parameter/feature	MCE-410 model	PMM 8057 w/EP-3B-01 probe
Type of probe(s)	E-field probes, isotropic, tri-axial	E-field probes, isotropic, tri-axial
Frequency response/range	0.1–3000 MHz, 900–3000 MHz	0.1–3000 MHz, 0.1–862 MHz, 933–3000 MHz
Physical quantity	Electric field strength, E(V/m)	Electric field strength, E(V/m)
Measurement range	0.5–130 V/m	0.2–200 V/m
Measurement resolution	0.1 V/m	0.01 V/m
Sensitivity	0.5 V/m	0.2 V/m
Temperature sensitivity	0.04 dB/°C	0.1 dB/°C
Measurement uncertainty	Total uncertainty (manufacturer) ± 2.5 dB	Total uncertainty (*k* = 1.96) ± 1.96 dB
Averaging interval	2–10 min (6 min is selected)	30 s–15 min (6 min is selected)
Sampling frequency	20 measurements/min	20 measurements/min
Result value	RMS, max, avg (RMS during 6 min is selected)	RMS, max, avg (RMS during 6 min is selected)

In^6^ more detailed information about the system architecture and sensor characteristics of the two networks can be found, together with a comprehensive uncertainty budget evaluated specifically for the PMM8057 with EP-3B-01 probe sensors. The measurements of fixed sensors in the two networks have been validated at the time of their installation. The total power flux density they measured was compared to that obtained with a frequency-selective measurement, which involved a spectrum analyzer. The percentage difference between the two values was always smaller than the root-sum-square of the uncertainties of the two measuring systems, fixed sensor and frequency-selective, the latter having an expanded uncertainty of less than 2.85 dB.

### Sensors installation locations and heights

The selected sensors are installed in twenty different regions in Northern Greece. Among these, seven sensors are installed in the densely populated urban area of the municipality of Thessaloniki, which has a population of 325,000 residents. Fourteen sensors are installed in urban areas with populations ranging from 52,000 to 165,000 residents. Additionally, seven sensors are installed in suburban areas with populations ranging from 10,000 to 43,000 residents, while three sensors are installed in rural areas with populations below 10,000 residents. Typically, these sensors are placed on the rooftops or balconies of buildings ranging from the 1st floor (3 m) up to the 6th floor (18 m). Out of the 31 sensors, two are installed on the fences of schoolyards (2 m), and four are placed on the roofs of prefab buildings (2.5 m).

The purpose of this work was to study the long-term temporal changes in the environmental EMF. Therefore, it is expected that these changes will always have the same impact on the mean power flux density value resulting from the measurements of all sensors. On the other hand, when the spatial distribution of environmental EMF or short-term changes are of interest, the height of the fixed sensors and the population density (which reflects the traffic of cellular networks) become important^2^.

### Data selection

The monitoring sensors had been installed outdoors, at different locations, on various dates, and for different operation intervals. In addition, many sensors might have been temporarily uninstalled, due to faults or for calibration. As a consequence, there is a large amount of data that is not uniform. In the present study, the data were aggregated into monthly intervals. The median value of the 6-min recordings was calculated for each month for each monitoring station (Fig. 1b). Therefore, for a monitoring sensor without data loss, twelve (12) values were obtained for each year. In case of missing values, these were replaced by the mean value of all 6-min data entries at the same location (the monitoring station was fixed at this location). Since the data collection was not uniform, it was necessary to select monitoring sensors at specific locations, using a group of criteria. We selected locations where sensors had been installed for more than 9 years, and they also had data loss in the range of 14–49% in the period from the end of 2003 until February 2022. The above procedure generated a dataset of 31 measuring sensors (17 sensors in the Hermes program and 14 in Pedion24).

**Figure 2:**
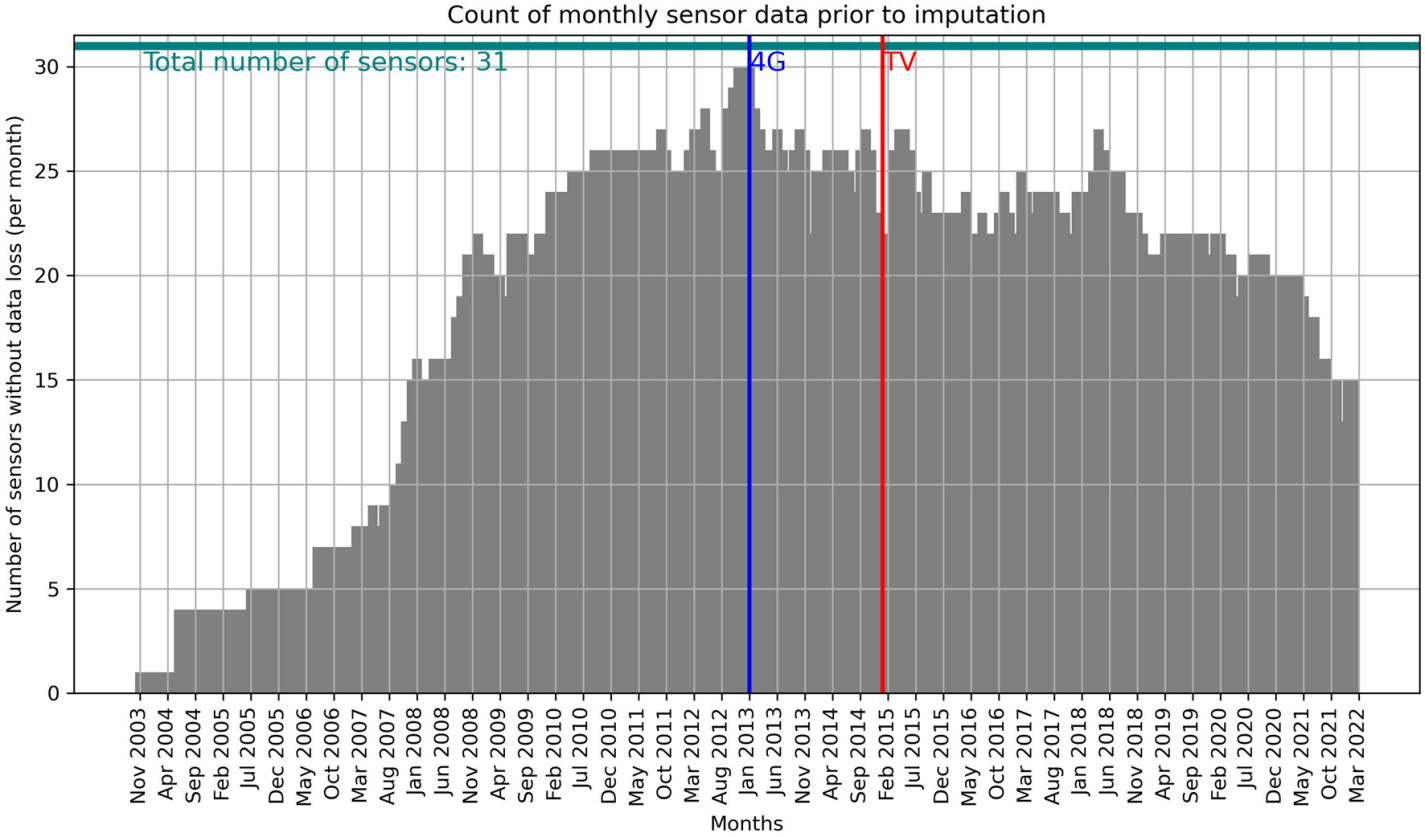
The number of sensors that contribute to the total mean value per month without data loss for a specific month. The blue and red lines depict the starting points of commercial use of 4G networks (blue) and the spectrum reallocation from analogue TV to digital TV and 4G in 700–800 MHz frequency band.

Figure 2 illustrates the number of sensors, for each month within the selected time interval, that contribute to the total mean value per month without experiencing data loss during that specific month. It is worth noting that the most significant portion of data loss occurred during the initial years (2004–2007) of network operations, which coincided with the establishment of numerous new installation locations. From October 2008 to April 2021, each month had at least 19 out of 31 sensors that provided their data without any loss. On average, 23.9 sensors (with a median of 24) contributed their actual data each month. This time span includes a key period in the current work: the transition from 3 to 4G cellular networks and the frequency reallocation at the 700–800 MHz band. The latter involved releasing spectrum from analogue TV and allocating it to 4G networks during the shift from analogue to digital TV (digital dividend).

### Data processing

First, we collected the 6-min raw data from the wideband filters (Fig. 1a) and aggregated them by calculating the median value per month for each location. This resulted in 12 values per year, one per month, at each location. Subsequently, we calculated the mean value for each month by aggregating the above median values from all locations. For instance, to obtain the value for January 2010, we calculated the mean value derived from the 31 location values for the same month. This process was repeated for every month across all years (Fig. 1b). The same procedure was executed for both high-pass (Pedion24, Hermes) and low-pass (Pedion24) filters. The metric collected and processed was the power flux density S (mW/m^2^) magnitude. Figure 1c illustrates the framework of analysis and the parameters-quantities examined in this study. In order to identify temporal trends in the data we used the STL method (Seasonal and Trend decomposition using LOESS).

The STL method^7^ has been widely utilized as a decomposition technique in various fields of science, including economics, biology, and communications. It has been employed to decompose time series into different components, thereby facilitating the identification of trends, seasonal patterns, and drawing meaningful conclusions. STL offers several benefits as it can detect time-varying seasonal components, effectively handle nonlinear trends, and exhibit robustness^8–10^ even in the presence of outliers making our choice for decompose and analysis of our examined timeseries.

The STL method decomposes a time series $Y_{v}$, into three components: a trend component $T_{v}$, a seasonal component $S_{v}$, and a residual component $R_{v}$, such that $Y_{v} = T_{v} + S_{v} + R_{v}$ for *v *= 1–*N*. The decomposition is done using locally fitted regression models to estimate the trend and seasonal components. The residual component is obtained by subtracting the estimated trend and seasonal components from the original time series. The STL method uses a sliding window to divide the data into smaller segments, and LOESS (Locally Estimated Scatterplot Smoothing) uses a polynomial fit of degree *d* in each segment. To perform the STL decomposition, the seasonality (12 for our monthly data) must be known. In this study, we used LOESS with locally-linear fitting (*d* = 1) implemented in the Python ‘Statsmodels’ module^11^. (More details on the implementation of the STL method and the parameters used can be found in the Supplementary Material of the manuscript available online.)

### Data grouping

The initial aim of the work was to analyze the time series data of all 31 sensors from the two monitoring networks. However, there is a major difference in the deployment of the two networks. Hermes monitoring sensors are typically installed on public buildings (e.g., schools, town halls) without necessarily having any base transceiver station (BTS) in their vicinity. On the contrary, Pedion24 sensors are mostly located close to a BTS. For this reason, we decided to process the monitoring sensors of the two projects (Pedion24 and Hermes) both as separate datasets, as well as an aggregated dataset. Thus, we analyzed the time series data using wideband and highpass filters for three different datasets: the entire dataset of 31 stations, the dataset of 17 sensors from Hermes, and the dataset of 14 sensors from Pedion24. The same analysis was performed for the lowpass filter data of Pedion24 sensors. The rationale behind the separate analysis was that the sensors in the vicinity of BTS not only record higher EMF values but also likely exhibit different variations in the EMF field, especially during periods when a new cellular technology is introduced (e.g., 4G) or when there is reallocation of spectrum by assigning new frequencies to cellular networks. To justify our decision for the separate analysis, we used the Spearman's rank correlation coefficient^12^ as a method to measure the association between the aforementioned datasets.

### Exploration of additional parameters

The large time span of recorded data led us not only to decompose the time series but also to explore additional parameters that will contribute to the knowledge of EMF behavior through the years.

#### Seasonality

To gain insights into the data's seasonal patterns, we analyzed the monthly data from various locations, using the data from the wideband filters. We calculated the average value for each month over an 18-years period, spanning from 2004 to 2022. This allows us to examine if there are months that systematically record higher values, providing a better understanding of the data's seasonality.

#### Maximum variability

Following a different approach from Joseph and Verloock^13^, who studied the weekly variation of the electric field, and Manassas et al.^14^, who studied the diurnal variability, we study the maximum variability of power density during the whole lifetime of the monitoring networks. Initially, we calculated the mean power density (S) per month, from all the sensors in each of the three categories (all sensors, Hermes, Pedion24) and for different filters. We calculated maximum variability $V\left( \% \right)$ as:

1 $$ V\left( \% \right) = \frac{{S_{max} - S_{min} }}{{S_{min} }} \times 100\left( \% \right) $$

where $S_{max}$ is the highest power density in a specific month between the years 2004 and 2022 and $S_{min}$ the lowest. We analyze this metric to estimate the maximum changes in EMF that can occur over a wide time span, aiming to attribute it to changes in wireless communications technology transitions.

### Ethical approval

All authors have read, understood, and have complied as applicable with the statement on "Ethical responsibilities of Authors" as found in the Instructions for Authors.

## Results and discussion

### Wideband filters

**Figure 3:**
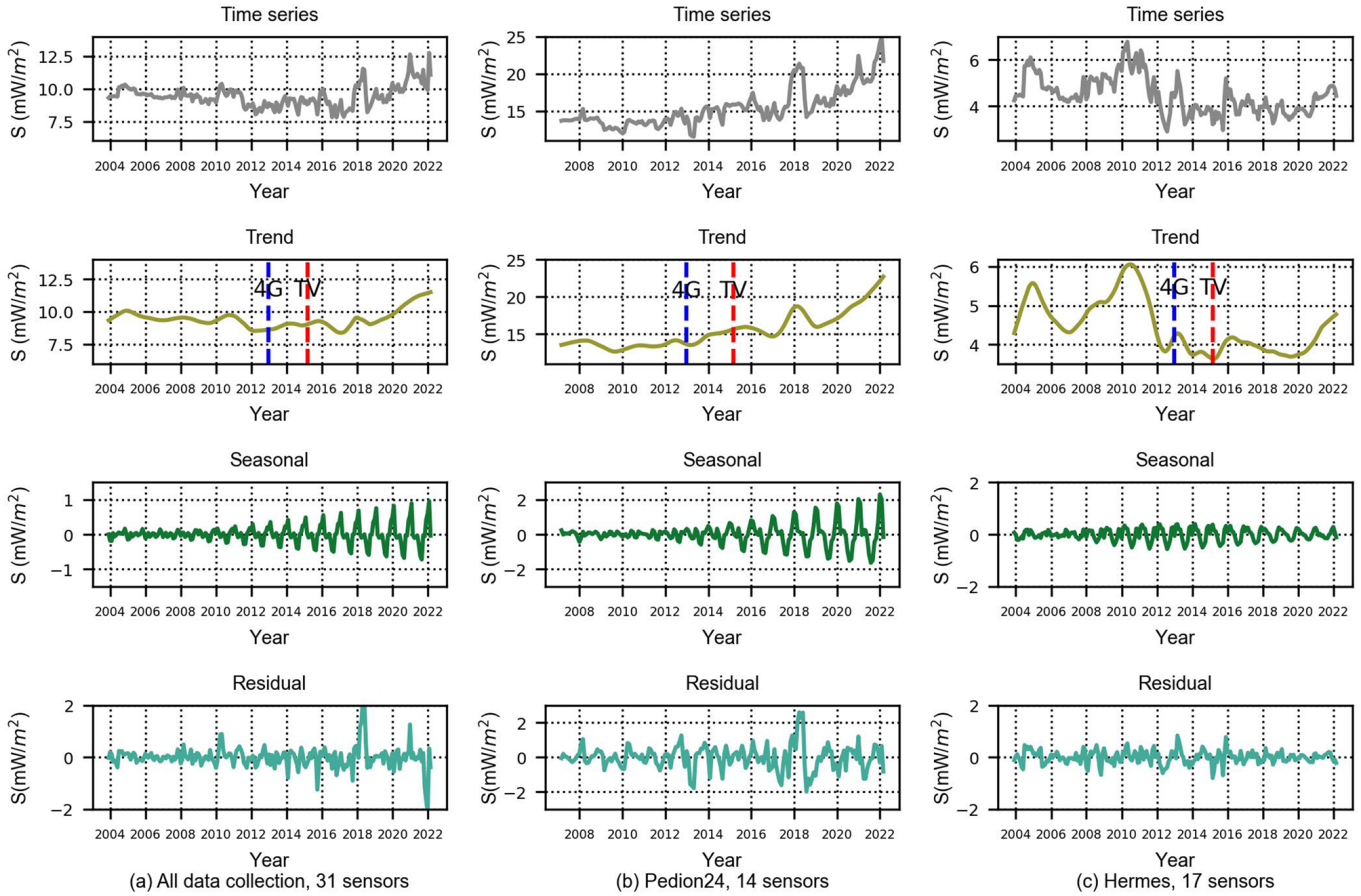
Timeseries and decomposition in trend, seasonal and residual components for the wide-band filters.

**Figure 4:**
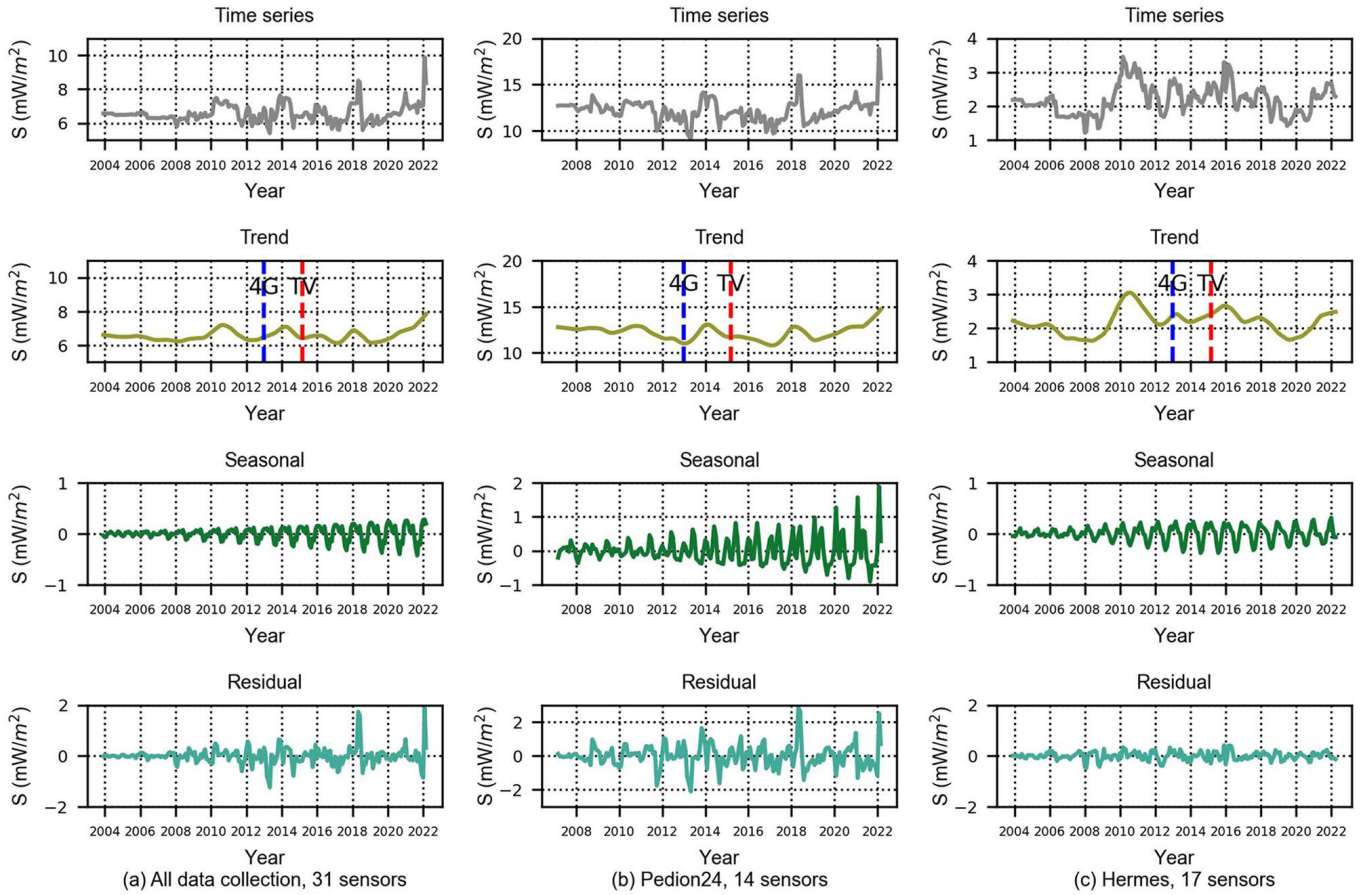
Timeseries and decomposition in trend, seasonal and residual components for high-pass filters.

**Figure 5:**
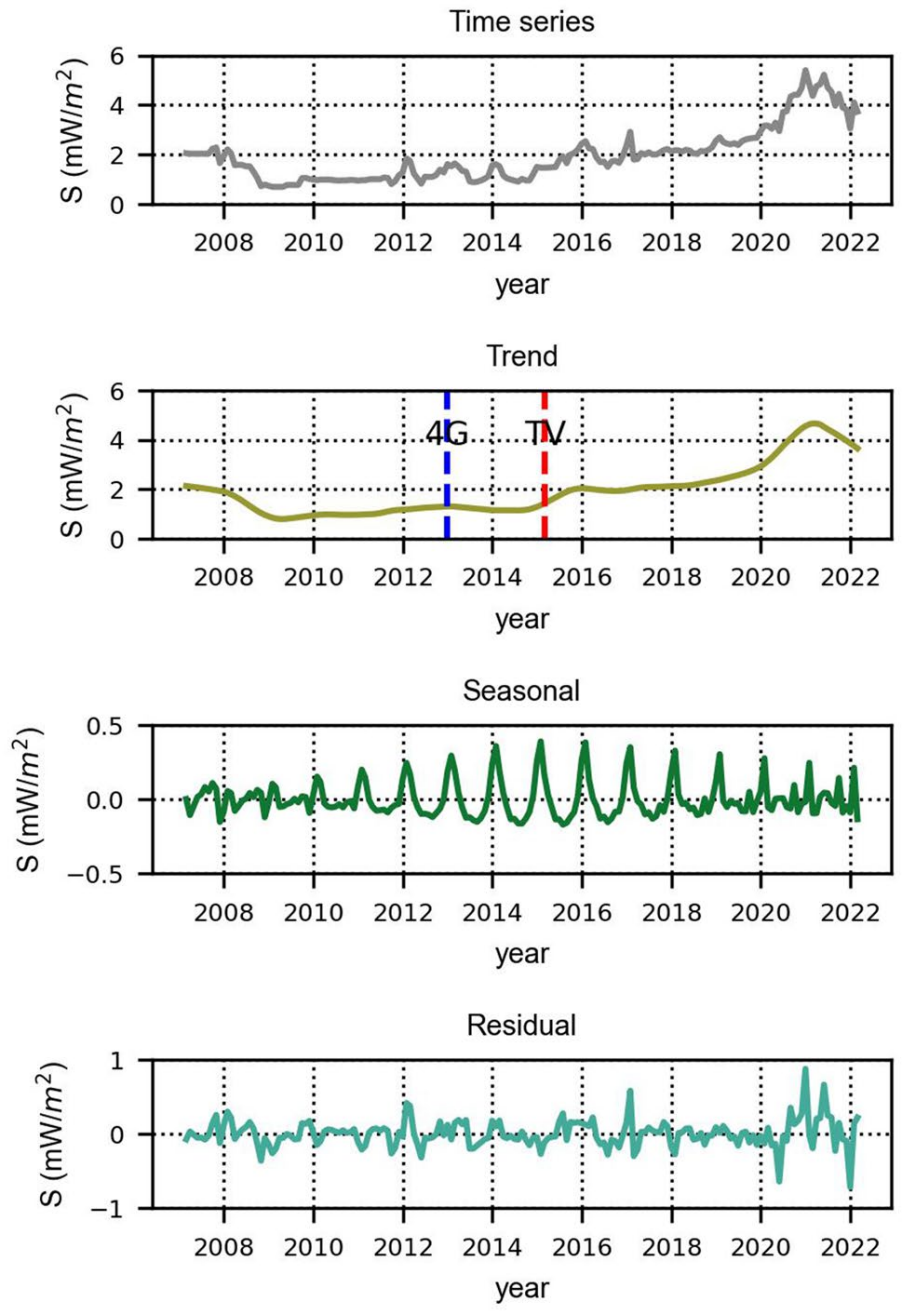
Time series and decomposition in trend, seasonal and residual components for 14 stations (Pedion24, low-pass filter).

Initially, we processed the grouped data of 31 sensors using the data of their wideband filters (Fig. 3a). However, due to the different installation strategies regarding the selection of locations for the two networks, it became apparent that the monitoring sensors from the Pedion24 and Hermes projects should also be processed separately (Fig. 3b,c). Figures 3, 4, and 5 depict the time series and their decomposition into trend, seasonal and residual components using the STL method, separately for the wideband, high-pass, and low-pass filters. In the trend graphs, the blue and red lines represent the starting points of commercial use of 4G networks (blue) and the spectrum reallocation from analog TV to digital TV and 4G in the 700–800 MHz frequency band (red). These two changes in the EMF environment are examined in relation to the resulting changes in EMF exposure.

As shown in Fig. 3a, the RF-EMF fluctuates over time, as indicated by the time series and corresponding trend. Focusing on the year 2017 and after, there is a discernible upward trend. As demonstrated in Fig. 3b (Pedion24 locations) and Fig. 3c (Hermes locations) it looks like this increasing trend can be attributed to Pedion24. The contribution of 14 locations of Pedion24 to the total 31 locations field is higher than the 17 Hermes locations. As referred previously, the difference between the two monitoring networks is that Hermes sensors are usually installed in public buildings without necessarily the existence of any BTS in their vicinity. On the contrary, Pedion24 sensors are mostly located close to a BTS. Therefore, the increasing trend after 2017 can be attributed to the transmitting power of the BTS (Pedion24 locations), although this is not reflected in the full electromagnetic environment (Hermes locations). In Fig. 3b (Pedion24) an upward trend is also obvious in 2013–2015. It is notable to mention that in 2013 the commercial use of the 4G network was launched in Greece. The differences between the trend patterns of Figs. 3b (Pedion24) and 3c (Hermes) after 2012, will be further analyzed below.

### High-pass filters

The same statistical processing was performed for the high-pass filters (Fig. 4). The Hermes program sensors are equipped with a high-pass filter in the range of 900 MHz–3 GHz and those of Pedion24 in the range of 933 MHz–3 GHz. The downlink frequencies in the GSM-900 band start at 925 MHz in Greece. All mobile telephony downlink frequencies were operating in the high-pass frequency range of the sensors until 2014. At the end of 2014, new frequencies below 900 MHz were allocated for 4G/LTE networks. As shown in the trend graph of Fig. 4a, during the adoption of 4G networks in Greece (mid-2013 till early 2014) there is an increase in power density, of less than 20%. The trend pattern of Fig. 4b confirms the fact of an increasing trend of environmental EMF in cellular frequencies after 2017.

It is worth noting that, as anticipated, the cumulative values for both the wideband and high-pass filters in the Pedion24 project are higher than those in the Hermes project. This is due to the fact that the Pedion24 sensors are installed in close proximity to BTS. When comparing the monthly aggregated values depicted in Figs. 3, 4, and 5 to the strictest limit set forth in Greek legislation (1200 mW/m^2^)^15^, it becomes apparent that all of the values are well below these limits. It should be noted that these limits on power flux density are 60% of the ICNIRP^16^. Since it is not justifiable to compare monthly averaged values with the 6-min average reference level foreseen in the legislation, the maximum ever recorded value from the 31 sensors of the two monitoring networks was searched in the database. A maximum 6-min average value of 970 mW/m^2^ was identified, which 20% lower than the strictest reference level of 1200 mW/m^2^ (in the frequency range 10–400 MHz) of the Greek legislation.

### Low-pass filter

As mentioned above, Pedion24 sensors except for wideband and high-pass filters, also include a low-pass filter (100 kHz–862 MHz). The same data processing resulted in Fig. 5.

We observe that after 2014 there is a significant increase in EMF. This increase coincides with the launch of 4G spectrum allocation in the 800 MHz frequency band (downlink frequencies: 790–821 MHz, uplink frequencies: 832–862 MHz). The above spectrum had previously been used for analogue television and military applications. The first licenses were issued to telecom operators in 2014, and in 2015 the commercial use of the new frequency band began. Over the next years, the new network continued to expand. The utilization of the new spectrum has been increasing, resulting in a clear upward trend for the environmental EMF, until 2021. Focusing on the graphs of Pedion24, the first 3 years (2014–2017) the high-pass filter has a decreasing trend (Fig. 4b), the low-pass an increasing trend (Fig. 5), and the wideband (Fig. 3b) is almost stable until 2016, with a small decrease from 2016 to 2017. As mentioned, in the years from 2014 to 2017, spectrum refarming (analogue TV–4G) and the transition from analogue to digital television broadcasting took place. We conclude that the consistency of the wideband levels throughout this period is a result of higher cellular frequency bands counterbalancing the increase in the 800 MHz frequency band (4G).

### Correlation

**Figure 6:**
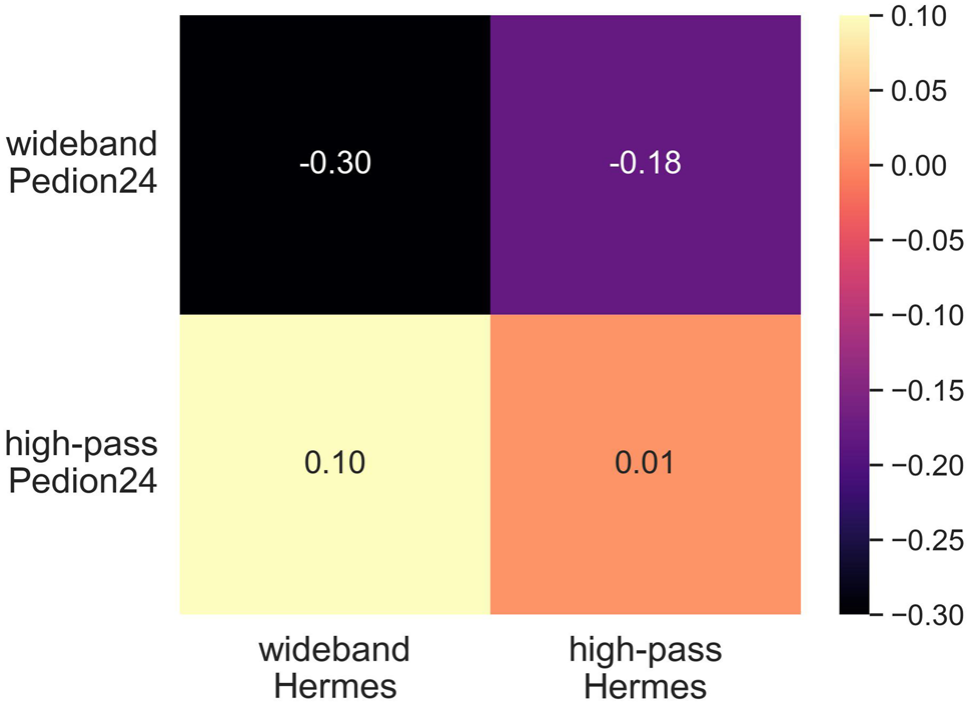
Spearman’s rho correlation coefficient between datasets.

In this paragraph, we use the Spearman’s rank correlation coefficient (rho value, ρ)^12^ as a method for measuring the association between the above datasets. Spearman’s rho correlation coefficient gives information about the degree of relationship between two datasets. It has a value between -1 to 1, with the value of 1 meaning perfect positive correlation and -1 perfect negative. A value of zero denotes no correlation. In Fig. 6 we summarize the results.

It is notable that there is no significant correlation between the high-pass filter values of Hermes and Pedion24 (ρ = 0.01). This can be attributed to the fact that Pedion24 sensors are typically installed near BTSs, whereas Hermes sensors are installed in random locations. The correlation with the corresponding data of their wideband filters is negative (ρ = − 0.30) thus indicating an inverse relationship between them. Upon examining the correlation between the datasets, we realized that it was necessary not only to process the grouping data of the two projects but also to conduct separate processing for each.

### Seasonality

**Figure 7:**
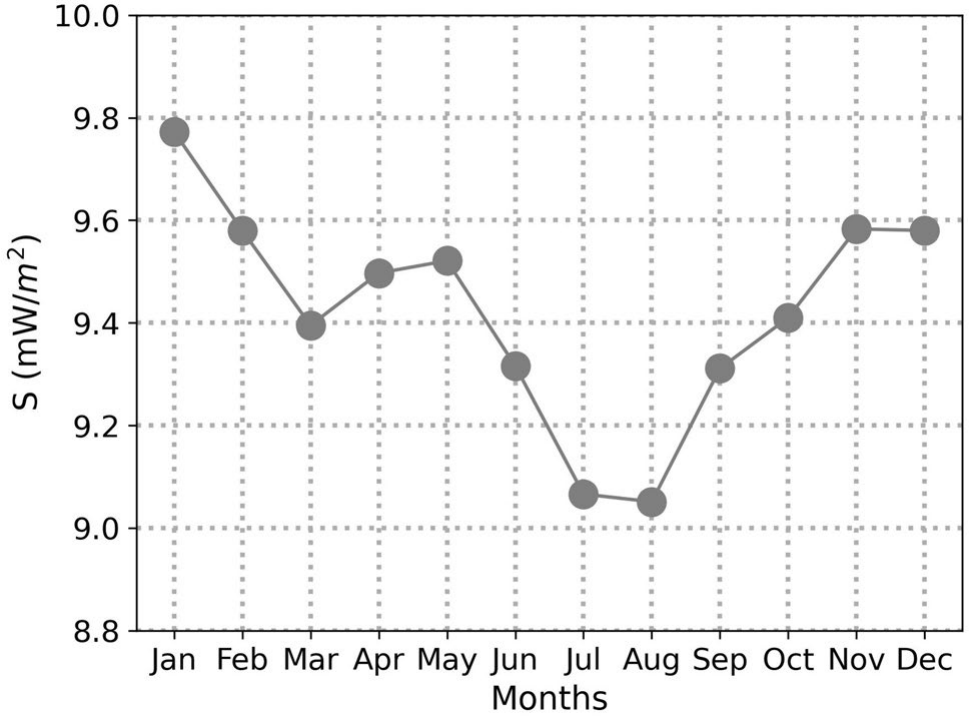
Monthly mean values for the period 2004–2022.

Upon analyzing the seasonal component of the graphs shown in Figs. 3, 4 and 5, it becomes evident that the data follows a yearly cycle in all cases. In order to better understand this trend, we have depicted the mean value per month using monthly data collection from all locations, specifically for the wideband filter, in Fig. 7. The value assigned to each month is calculated as the mean value of that month over the span of 18 years, ranging from 2004 until 2022. Interestingly, the recorded values are comparatively higher in the month of January.

Conversely, the months of June, July, August, and September exhibit lower measurements as compared to the other months. This can be attributed to the trend of residents moving from cities to resorts during this time of the year. The resultant decrease in the residential population in urban areas is directly responsible for the lower use of cellular networks, which, in turn, leads to a reduction in the recorded electric field values since the majority of measuring sensors are installed in these locations**.** This decrease is particularly noticeable during the peak vacation months of July and August, and contributes to the observed seasonal fluctuations in the collected data.

### Maximum variability

Using Eq. (1) we calculated the maximum variability. Table 2 presents the results of maximum variability for the three examined datasets and the corresponding filters.

**Table 2:** Maximum variability.

	Wideband filter (%)	High-pass filter (%)	Low-pass filter
All sensors (n = 31)	64	83	–
Pedion24 (n = 14)	85	107	713%
Hermes (n = 17)	134	183	–

It can be seen from Table 2 that the maximum variability of wideband and high-pass filters ranged between 64% (wideband filter—all locations) and 183% (high-pass filter—Hermes project locations). The highest level of variability, reaching 713%, is observed in the low-pass filter of Pedion24. Similarly, the steepest upward trend (Fig. 5) is observed for the same filter, after 2014. This can be attributed to the assignation of the 800 MHz spectrum to 4G systems after 2014, which resulted in significantly higher field values in that filter. This launch increased the field near base stations but, eventually, when we examined the Hermes monitoring sensors (at random locations) we found that there was an overall decrease in this band (see next paragraph “The contribution of different electromagnetic radiation sources to total exposure” section), mainly due to the transition from analogue to digital TV^17^.

The wideband and high-pass filters of Hermes sensors show greater variability than Pedion24 sensors. The diurnal variability^14^ resulted higher in the vicinity of BTS and this can be attributed to mobile phone networks operation, which causes fluctuation of the electric field between day and night. On the contrary, in this work, the long-term (maximum) variability resulted lower for sensors that are installed at locations close to a BTS compared to sensors at random locations.

### The contribution of different electromagnetic radiation sources to total exposure

Previous studies have shown that human exposure to EMF fields results mainly from mobile devices rather than mobile base station antennas. Gati et al.^18^ have shown that exposure due to the base stations is clearly much lower than exposure to mobile phones, but for 3G-UMTS measurements, the fast power control and the devices’ high sensitivity adapt the transmitted power so that the induced local exposure becomes considerably reduced compared to 2G systems. Following an integrative approach, Varsier et al.^19^ introduced the concept of global exposure index (EI), which considers both uplink (mobile devices) and downlink (base stations) exposures, as well as other environmental sources (e.g., exposure from WiFi routers or uplink from a bystander’s mobile device). They assumed that uplink contribution to whole-body specific absorption rate (SAR) was higher than downlink contribution in 2G and 3G cellular systems. This was also assumed in the study of van Wel et al.^20^, where it was further explained that 2G mobile phones contributed more than 3G devices to whole-body exposure. Huang and Wiart^21^ showed that the global EI reduced from 3 to 4G cellular systems, although the transmitted median power from mobile devices is similar for the two network generations^22,23^.

**Figure 8:**
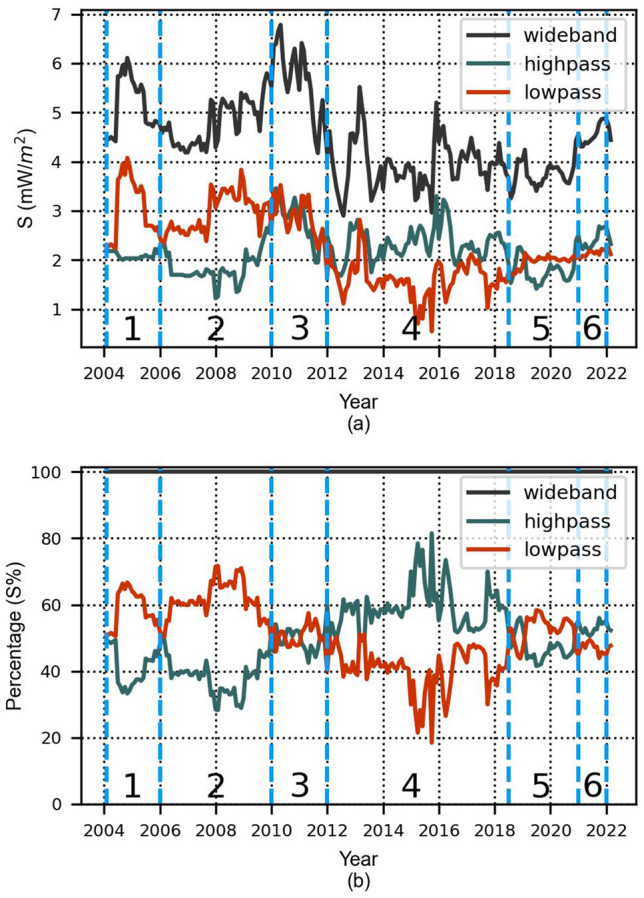
(**a**) Time series of wideband, high-pass, and lowpass frequencies, (**b**) time series of wideband, high-pass, and lowpass frequencies normalized to the wideband. Data originate from the 17 sensors of the Hermes monitoring network only. The vertical light blue lines and the numbers at the bottom depict the time intervals of Table 3.

In this section, we study the exposure to outdoor environmental EMF from transmitting sources, other than handheld mobile devices. The emissions from mobile BTS can be distinguished from those of all other sources (private radio, broadcasting, governmental services, etc.). The separation occurs due to the filters of the measuring sensors. Since the sensors of Pedion24 are located close to BTS, we can use the sensors of Hermes which are installed in random positions. As we mentioned above, Hermes stations contain only a wideband (100 kHz–3 GHz) and a high-pass filter (900 MHz–3 GHz). The power flux density in the frequency band 100 kHz–900 MHz was calculated as *S*_*low*-*pass*_ = *S*_*wideband*_ − *S*_*high*-*pass*_. We followed the same procedure as described above and we found the power flux density per month for this frequency band. In Fig. 8a, we distinguish the values recorded at Hermes locations in the wideband, high-pass filter, as well as those calculated in the low-pass frequency range.

To make it clearer which filter has the largest contribution to the overall field, the low-pass and high-pass values were normalized to the wideband values (Fig. 8b).

Figure 8 is divided into different time periods, as indicated in Table 3. The Table also displays the average percentage of the total power flux density allocated to the high-pass and low-pass frequencies for each time period. During those periods, the significant changes that contribute to EMF are discussed.

**Table 3:** Different time periods of Fig. 8 alongside the average contribution of the low and high-pass frequency bands and the main changes in EMF.

Time interval	(1) 2004–2006	(2) 2006–2010	(3) 2010–2012	(4) 2012-Mid 2018	(5) Mid 2018–2021	(6) 2021–2022
Low-pass average contribution	58.8%	61.4%	50.4%	40.7%	52.6%	47.0%
High-pass average contribution	41.2%	38.6%	49.6%	59.3%	47.4%	53.0%
Main changes introduced to the electro-magnetic environment	Not enough data from sensors to draw conclusions	Broadcasting dominates cellular transmissions	Introduction of digital TV together with analogue TV	Introduction of 4G networks /Digital dividend	Introduction of digital radio (DAB)	No further deployment of DAB

Figure 8 depicts that power flux density values in the low-pass filter are higher than the high-pass, during the interval 2004–2010 apparently because the broadcasting transmissions (FM, analogue TV) have higher contribution to the total field than cellular networks. Τhe reduction in low-pass after 2008–2009 coincides with the shutdown of unlicensed FM radio stations (88–108 MHz), in Greece.

In the time period 2010–2012 there is a brief increase of the low-pass filters’ contribution. In 2010, alongside the then existing analogue transmission, digital TV broadcasting was initiated. So, the small increase in 2010 can be attributed to the addition of digital broadcasting parallel to the analogue signal. In December 2012 the analogue signal switched-off for the commercial nationwide TV channels from the main broadcasting locations. In Fig. 8b we can observe that the raise that started in 2010 is followed by a decrease in late 2012.

From 2012 until 2018 EMF emissions are higher in the frequencies of the high-pass filter. In the same time period, the values in the low-pass filter decrease significantly, although in 2014 the 800 MHz frequency band was released for 4G network deployment. The reduction of power density in low-pass after 2014 may be attributed to the ongoing transition from analogue television to digital, which had started in late 2012. From the above, it can be observed that within the same frequency range (100 kHz–900 MHz), the combined exposure from cellular 4G networks and digital TV coincides with lower levels of exposure compared to analogue TV^17^.

During the years 2004–2014 the high-pass filter of Hermes sensors included all the downlink frequencies of cellular networks. For this period, we calculated the average contribution of the power flux density in high-pass (44.8%) and low-pass frequencies (55.2%) to the total field. The contribution of mobile cellular networks to the total field resulted lower than the other wireless applications. Over the following years after 2014, the low-pass filter includes the 4G band at 800 MHz. In the period 2014–2018 the average contribution of the high-pass frequencies to the overall recorded field was 60.9%. The respective contribution of the low-pass frequencies was 39.1%. We thus conclude that the contribution of cellular networks to the total field was higher than all the other wireless applications.

The increasing pattern in the low-pass filter after mid-2018 coincides with the beginning of the test period for DAB radio by Hellenic Broadcasting Corporation, the state-owned public radio and television broadcaster of Greece (ERT). In the mentioned period the ERT installed transmission equipment for DAB radio at the main broadcasting site of Thessaloniki. The analogue radio remained in operation. It is not captured when the test period ended since the DAB radio did not find widespread adoption in Greece. However, this increase in the low-pass filter frequencies after mid-2018, and the subsequent decrease in 2021, might also reflect a change in the architecture of the cellular networks with 4G BTS being deployed by the operators in the 800 MHz band, which were later (in 2021) substituted by 5G BTS in the NR FR1 band that was auctioned in Greece in December 2020.

The average normalized value (S%) for the period 2004–2022 is 50.4% for the low-pass frequencies and 49.7% for the high-pass.

## Conclusions

This study has emphasized the crucial importance of establishing continuous electromagnetic field (EMF) monitoring networks over a prolonged period to monitor compliance with safety limits for public exposure to electromagnetic radiation. These networks not only provide a means to ensure safety of the general public but also offer the potential to provide valuable information on spatial or temporal differences in exposure to environmental EMF, which may be useful for epidemiological purposes. The data obtained from such networks can be subjected to a detailed analysis to identify trends, patterns, and potential health risks associated with EMF exposure. However, the advent of new technological innovations, such as 5G base station antennas with advanced features, like beamforming and massive multiple-input-multiple-output (MaMIMO), poses significant challenges to the strategy for the deployment of such monitoring networks.

### Supplementary Information

Supplementary Information.

## Data Availability

This study used a part of datasets from publicly available data that are published in graphic forms on different subpages of the web pages: https://pedion24.gr/, http://hermes.physics.auth.gr/gr/main (accessed on 19 May 2023). The exact datasets analyzed during the current study are available from the corresponding author on reasonable request.
