# Supplementary material for: A study of the long term changes in the electromagnetic environment using data from continuous monitoring sensors in Greece
Source: Sci Rep. 2023 Aug 23;13:13784. doi: 10.1038/s41598-023-41034-3 (PMC10447458; doi:10.1038/s41598-023-41034-3)
Supplement: Supplementary file 1 — Supplementary Information. [file 41598_2023_41034_MOESM1_ESM.docx]

***A. STL decomposition method***

The STL method decomposes a time series $Y_{v}$, t = 1, 2, ..., n into three components: a trend component $T_{v}$ , a seasonal component $S_{v}$, and a residual component $R_{v}$, such that $Y_{v}=T_{v}+S_{v}+R_{v}$ for $v=1 to N$. The process of decomposition involves utilizing locally fitted regression models to approximate the trend and seasonal components. By subtracting these estimated components from the original time series, we obtain the residual component. The STL technique uses a sliding window to divide the data into smaller segments, while LOESS applies a polynomial fit of degree d to each segment. To perform the STL decomposition, the seasonality (12 for our monthly data) must be known. In this study, we used LOESS with locally-linear fitting (first-degree polynomial).

The STL algorithm involves two recursive procedures: an inner loop nested inside an outer loop, and relies on six important parameters:

$n_{(p)}$: the number of observations in each cycle of the seasonal component;

$n_{(i)}$: the number of passes through the inner loop;

$n_{(o)}$: the number of robustness iterations of the outer loop;

$n_{(l)}$: the smoothing parameter of the low-pass filter;

$n_{(t)}$: the trend smoothing parameter;

$n_{(s)}$: the seasonal smoothing parameter;

The inner loop fits the trend and computes the seasonal component. Each inner loop consists of six sequential steps:

1. Detrending. Compute the detrended series $Y_{v}-T_{v}^{(k)}$ for each $v=1 to N$, where k represents the loop step.

2. Smoothing of Cycle-subseries. Subdivide the time series into cycle-subseries $C_{v}^{(k+1)}$. Apply loess smoothing to each cycle-subseries with parameters $q=n_{s}$ and d = 1 (degree of the polynomial). This produces a temporary collection of smoothed values ranging from $v=-n_{\left( p \right)}+1$ to $N+n_{(p)}$.

3. Low-pass filtering of previous smoothed Cycle – subseries: Apply a low-pass filter to $C_{v}^{(k+1)}$ using a moving average of length $n_{(p)}$. Then, apply loess with parameters $d=1$ and $q=n_{(l)}$ to obtain the output series $L_{v}^{(k+1)}$ for v = 1 to N.

4. Detrending of smoothed Cycle – subseries: Detrend the smoothed cycle-subseries by subtracting the seasonal component from the $\left( k+1 \right)st$ iteration: $S_{v}^{(k+1)}=C_{v}^{(k+1)}-L_{v}^{(k+1)}$ for v = 1 to N.

5. Deseasonalizing: Deseasonalize the series by subtracting the seasonal component: $Y_{v}-S_{v}^{(k+1)}$.

6. Trend smoothing. Smooth the series trend using loess with parameters $q=n_{(t)}$ and $d=1$. The trend component $T_{v}^{(k+1)},$ for each v = 1 to N is obtained from the smoothed values computed at all time positions.

In the event of detecting any anomalies, an outer loop will be employed to replace the LOESSs at the second and sixth steps of the inner loop with the robust LOESS method, by computing and adjusting robustness weights $p_{v}$ for $n_{(o)}$ times that captures the extremity of the resulting remainder value $R_{v}$. Initially, $p_{v}$ is set to 1, but it is updated as more precise approximations of $R_{v}$are obtained. The inner loop results in the estimates of $T_{v}$ and $S_{v}$ for the trend and seasonal components. Then the reminder component is calculated $R_{v}=Y_{v}-T_{v}-S_{v}$. The robustness weight at time point $v$ is determined using the bisquare weight function B(u): $p_{v}=B\left( \frac{\left| R_{v} \right|}{h} \right)$. The parameter h accounts for outliers and is calculated as $h=6\cdot median\left( \left| R_{v} \right| \right)$ and B is the bisquare weight function:

$$B\left( v \right)=\left\{ \begin{aligned} \left( 1-u^{2} \right)^{2}, 0\leq u<1 \\ 0, u>1 \end{aligned} \right.$$

***B. STL parameters selection***

The selection of the value for $n_{\left( p \right)}$ depends on the expected periodicity of the data. For monthly data, with a yearly periodicity, $n_{\left( p \right)}$ is set to 12. For $n_{\left( i \right)}$, while in many cases, $n_{\left( i \right)}=1$ is sufficient, using $n_{\left( i \right)}=2$ provides near certainty of convergence. If robustness iterations are required, $n_{\left( o \right)}$ should be set to a value other than zero. In such cases, the $n_{\left( o \right)}$ should be set to a non-zero value, chosen large enough for robust estimates of the trend and seasonal components to converge. For $n_{\left( l \right)}$ the value can be chosen as the smallest odd integer greater than or equal to $n_{\left( p \right)}$. The value of $n_{\left( s \right)}$ should always be an odd number, with a minimum value of 7. Lower values (e.g., ranging from 7 to 10) promote the utilization of localized data, whereas higher values aggregate values from the corresponding time of the year throughout the time-series. Regarding $n_{\left( t \right)}$, it should be selected to satisfy the following condition: $n_{\left( t \right)}\geq\frac{1.5n_{\left( p \right)}}{1-1.5n_{(s)}^{-1}}$ . Typically, $n_{\left( t \right)}$ ranges from about 1.5$n_{\left( p \right)}$ to 2$n_{\left( p \right)}$. A good practice is to select the smallest odd integer that satisfies the previous equality.

In the present study the following parameters were used for the STL decomposition. The first column of the next table (Table S1) shows the parameters following the notation presented previously and used in the original paper (Cleveland et al., 1990). The second column presents the selected values of the parameters. The values were selected carefully following the previous mentioned criteria and by studying the examples of the original paper (Cleveland et al., 1990) that gives sufficient examples using periodic monthly data.

Table S1. Values of parameters used for STL decomposition

| Parameter | Value |
| --- | --- |
| $n_{\left( p \right)}$ | 12 |
| $n_{\left( i \right)}$ | 2 |
| $n_{\left( o \right)}$ | 15 |
| $n_{(l)}$ | 13 |
| $n_{(t)}$ | 19 |
| $n_{(s)}$ | 35 |
